# Action of Bromide of Potassium

**Published:** 1881-01

**Authors:** 


Action of Bromide of Potassium.—Maragliano has found by employing the method of cranial thermometry, that bromide of potassium, in doses of thirty to fifty grains, contrary to the usual theories of its action, causes a rise of temperature, at least on the outside of the cranium. This rise amounts on the average to about one degree centigrade; it reaches its acme in about an hour and a half and declines again in two or three hours. Simultaneously with this there appears a slight rise of two or three tenths of a degree in the exilla. It is of course open to question, especially after the publication of Franck's researches, whether the temperature or the changes of temperature on the external surface of the head, represent approximately similar conditions of the brain or not. There does, however, seem to be some clinical evidence that there is a connection between external cranial temperature and intra-cranial changes, and if this rise following the ingestion of bromides really means an increase of cerebral circulation, then a popular theory of its action will have to be given up—*Chicago Med. Review.*

